# Novel regulatory mechanism of choline-O-sulfate and choline catabolism by two BetIs in Alphaproteobacteria

**DOI:** 10.1128/aem.00333-25

**Published:** 2025-08-13

**Authors:** Jia-Rong Liu, Zhen-Kun Li, Ming-Chen Wang, Na Wang, Zhi-Qing Wang, Fei-Fei Li, Yin Chen, Yu-Zhong Zhang, Hui-Hui Fu

**Affiliations:** 1 MOE Key Laboratory of Evolution and Marine Biodiversity, Frontiers Science Center for Deep Ocean Multispheres and Earth System, College of Marine Life Sciences, Ocean University of China 12591, Qingdao, China; 2 State Key Laboratory of Microbial Technology, Marine Biotechnology Research Center, Shandong University 12589, Qingdao, China; 3 Laboratory for Marine Biology and Biotechnology, Qingdao Marine Science and Technology Center and Laoshan Laboratory 474988, Qingdao, China; 4 School of Life Sciences, University of Warwick 117213, Coventry, United Kingdom; Washington University in St. Louis, St. Louis, Missouri, USA

**Keywords:** choline-O-sulfate, choline, BetI, regulatory mechanism, Alphaproteobacteria

## Abstract

Choline-O-sulfate (COS) and choline are ubiquitous in the environment, and diverse bacteria catabolize them into glycine betaine for osmoprotection or as a carbon and/or nitrogen source. The characterized *bet* genes involved in COS and choline catabolism are usually clustered in the genome with one regulatory gene, *betI*. Here, we report a novel regulatory mechanism of COS and choline catabolism by two BetIs in the model marine Roseobacter group bacterium *Ruegeria pomeroyi* DSS-3. The insertion of two unrelated genes divided the *R. pomeroyi* DSS-3 *bet* cluster into two parts, with each part having its own regulatory *betI*. BetI1 deregulates the transcription of the *betI1-betC* operon and *betB* in the presence of choline. COS and choline induce the transcription of the structural genes while repressing the regulatory gene of the *betI2-betTA* divergon. Two palindromes with one shared flanking sequence in the intergenic fragment of this divergon are recognized by BetI2. The affinities of BetI2 to these two *betI2 boxes* are fine-tuned by the binding of the effector choline. Bioinformatic analysis indicated that two *betIs* exist widely in members of Alphaproteobacteria. This study elucidates a novel regulatory pattern of COS and choline catabolism in abundant bacteria.

**IMPORTANCE:**

Choline and its sulfonium analog choline-O-sulfate (COS) are ubiquitous, and their catabolism by the bacterial choline-to-glycine betaine pathway generates a potent osmoprotectant, glycine betaine, and also provides carbon and nitrogen sources. In contrast to previously characterized modes executed by one regulatory BetI, in this study, we elucidate a novel regulatory mechanism of COS and choline catabolism by two BetIs in the model marine Roseobacter group bacterium *Ruegeria pomeroyi* DSS-3. The two BetIs control distinct steps of COS and choline catabolism and respond differently to osmotic stress. This study indicates that the two BetIs regulatory mode is a long-overlooked mechanism adopted by abundant bacteria.

## INTRODUCTION

Choline-O-sulfate (COS) is widely distributed in marine microalgae and plants in the form of phosphatidylsulfocholine, the sulfonium analog of the membrane lipid phosphatidylcholine (1–5). COS and choline can be liberated from their corresponding phospholipids through the action of phosphodiesterases, which are widely present in plants, viruses, bacteria, fungi, and animals (6). Liberated COS can function as an osmoprotectant in some plants (2, 5, 7) and bacteria (8). COS can also be converted into choline by the choline sulfatase BetC (8). Choline is the precursor of glycine betaine (GBT), one of the most potent osmoprotectants (9, 10).

**Fig 1:**
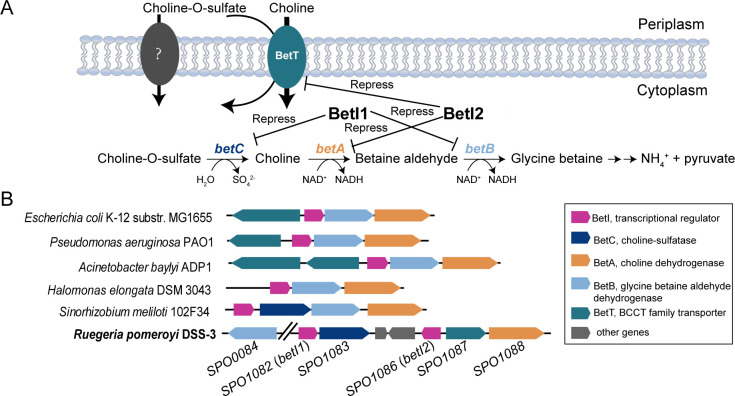
(**A**) Proposed pathway of choline-O-sulfate catabolism and the regulatory roles of two BetIs on *bet* genes. BetT, choline and choline-O-sulfate transporter; *betC* encodes choline sulfatase; *betA* encodes choline dehydrogenase; and *betB* encodes betaine aldehyde dehydrogenase. (**B**) Organization of *bet* genes in representative bacteria. *Ruegeria pomeroyi* DSS-3 studied here is shown in bold with the gene loci indicated below.

Diverse bacteria, plants, and animals harness the choline-to-glycine betaine pathway for osmoadaptation (11–16) and the utilization of COS and choline as carbon and nitrogen sources (17–24). The two-step choline-to-glycine betaine pathway is mediated by choline dehydrogenase BetA and betaine aldehyde dehydrogenase BetB (Fig. 1A), which have been characterized in diverse organisms, including bacteria and plants (15, 25–31). Choline catabolic genes are usually clustered in the genome, termed the *bet* cluster. Apart from *betA* and *betB*, two other structural genes in the *bet* cluster are *betT* and *betC. betT* encodes the choline transporter and usually forms a divergon structure with the *betBA* operon, sharing the same promoter region. *betC* in *Sinorhizobium meliloti* is in the *betICBA* operon (8). While *Pseudomonas* spp. *betC* genes are located well away from *betBA* (32, 33), the regulatory gene *betI*, encoding a TetR family transcriptional repressor, is located in the *bet* cluster as well. It is usually co-transcribed with *betBA* in diverse bacteria (8, 24, 26, 34–37).

Regulation of the choline catabolism/*bet* clusters can vary in different bacteria. The *Escherichia coli bet* cluster is regulated by oxygen, osmotic stress, and BetI, for which the binding of the inducer choline stimulates the BetI-DNA complex formation (34, 38). The transcript level of *betBA* in *Pseudomonas aeruginosa* is induced by choline mediated by BetI, in addition to osmotic stress (37). In addition, its choline transporter BetT is upregulated by hyperosmotic stress (39), and its structure and mechanism in response to osmotic stress have been reported recently (40). *Pseudomonas* spp. *betC* is regulated by the LysR family regulator BetR and is not required for osmoprotection (32). The *betIBA* operon in *Acinetobacter baylyi* is regulated by choline-responsive BetI, osmotic stress, and quorum-sensing regulator AnoR (36). While one of the two divergently transcribed *betTs* is osmo-dependent, the other is osmo-independent (17, 23). The *S. meliloti betICBA* operon is induced by choline, COS, and acetylcholine but not by osmotic stress (41).

As one of the most abundant groups in the marine environment, the marine Roseobacter group bacteria (MRB), a monophyletic group of Alphaproteobacteria, play a major role in carbon, sulfur, and nitrogen cycling. The model MRB bacterium *Ruegeria pomeroyi* DSS-3 can grow on COS and choline as a carbon or nitrogen source, and the structural genes in the *bet* cluster have been characterized previously (20). However, the organization of the *bet* cluster in *R. pomeroyi* DSS-3 is markedly different from those characterized in other bacteria: (i) the insertion of two unrelated genes in the *bet* cluster; (ii) *betB* is located out of the *bet* cluster; and (iii) the presence of two *betI* genes, while all previously reported *bet* clusters only have one *betI* (Fig. 1B). The roles of these two BetIs in regulating *bet* genes are unclear. This study elucidates the regulatory mechanism of these two BetIs and demonstrates that the regulation of the *bet* cluster by two BetIs is widespread in Alphaproteobacteria.

## MATERIALS AND METHODS

### Bacterial strains and growth conditions

The bacterial strains and plasmids used in this study are listed in Table S1. Routinely, *Escherichia coli* strains WM3064 and BL21(DE3) were grown in Lysogeny broth (LB) medium at 37°C. *R. pomeroyi* DSS-3 was grown in marine broth 2216E (Difco) medium at 30°C for genetic manipulation. When appropriate, the growth medium was supplemented with the following: gentamicin, 15 µg/mL; 2,6-diaminopimelic acid, 0.3 mM; kanamycin, 50 µg/mL; and ampicillin, 50 µg/mL. To determine growth on COS as a nitrogen source, *R. pomeroyi* strains were grown in marine ammonium mineral salts (MAMS) medium (20, 42, 43) with (NH_4_)_2_SO_4_ replaced by COS (2 mM) and glucose (10 mM) as the carbon source. Salinity tolerance of *R. pomeroyi* DSS-3 was determined with supplementation of 3%–7% (wt/vol) NaCl as previously described (44).

### Mutagenesis and complementation

The *att*-based fusion PCR method previously described (45) was used to construct in-frame deletion strains of *R. pomeroyi* DSS-3 with moderate modifications using specific primers (Table S2). Briefly, the fusion of two fragments flanking the target gene was introduced into the *att*-based mutagenesis plasmid pHGM01 by site-directed recombination and transformed into *E. coli* WM3064. The resultant plasmid was transferred into recipient strains via conjugation. In-frame deletion mutants were screened and verified by PCR sequencing (45). For complementation of gene mutants, fragments containing the target genes and their native promoters were amplified by PCR and cloned into pHG101 (46) using specific primers (Table S2). The resulting complementation vectors were transferred into host bacteria by conjugation via *E. coli* WM3064 and verified by PCR and sequencing. The primers used for complementation are listed in Table S2.

### Real-time qPCR analysis and reverse transcription PCR

Bacteria were precultured in 2216E medium at 30°C to an OD_600_ of 0.6. Cells for total RNA extraction were prepared by inoculating cultures in MAMS medium with 2 mM NH_4_^+^, COS, or choline as sole nitrogen source for 4 hours. Total RNA was extracted using the RNeasy Mini Kit (Qiagen, Germany). Real-time qPCR was performed with specific primers (Table S2). For reverse transcription PCR, the reverse-transcribed cDNA of *R. pomeroyi* DSS-3 grown with 2 mM COS was used as the PCR template with specific primers (Table S2). The *R. pomeroyi* DSS-3 genome was used as a control.

### Protein purification

*betI1* and *betI2* were amplified from the *R. pomeroyi* DSS-3 genome and cloned into pET28a (+) vector with a C-terminal His-tag (Novagen, USA). BetI1 and BetI2 proteins were overexpressed in *E. coli* BL21(DE3). Cells were cultured in LB medium at 37°C to an OD_600_ of 0.8–1.0 and then induced at 16°C for 14 hours with 0.4 mM isopropyl-β-D-thiogalactopyranoside. After induction, cells were collected by centrifugation, resuspended in PBS buffer, and lysed by pressure crusher. Proteins were purified using a Ni^2+^-NTA column (GE Healthcare, USA), followed by gel filtration on a Superdex G200 column (GE Healthcare, USA) as described by Shao et al. (47).

### DNase I footprinting assays

The promoter region of *betI1* was cloned into plasmid pGEM-T (Promega, USA) and amplified using T7 (FAM) and SP6 primers to generate a fluorescent FAM-labeled probe. Fluorescent probes were purified and quantified with NanoDrop 2000C (Thermo Fisher Scientific, USA). DNase I footprinting assays were performed according to Wang et al. (48). For each assay, 400 ng of probes were incubated with 2 µg of BetI1 or without it in a total volume of 40 µL. Phenol/chloroform and ethanol were used to successively extract and precipitate samples. The obtained samples were then dissolved in 30 µL MiliQ water. DNA ladder preparation, electrophoresis, sequencing, and data analysis were performed as described in Wang et al. (48). The GeneScan-LIZ600 size standard (Applied Biosystems) was used.

### Electrophoretic mobility shift assays

Electrophoretic mobility shift assays (EMSAs) were performed in 20 µL reaction mixtures containing different concentrations of BetI1 (0–28 μM) or BetI2 (0–28 μM) and various DNA probes (1 nM, except for probes P_29_ and P_30_) in binding buffer (2 mM EDTA, 20 mM KCl, 0.5 mM DTT, and 4% Ficoll-400, pH 8.0) with 2 µg poly (dI-dC) as unspecific competitor (49). DNA probes P*_betI1C_*, intergenic region (IGR), and P*_betB_* were amplified from *R. pomeroyi* DSS-3 genomic DNA using specific primer sets, with forward primers 5′-biotin-labeled (Table S2). DNA probes P_29_ and P_30_ were obtained by annealing the complementary oligonucleotides of the 29 bp BetI1-protected region of P*_betI1C_* and the 30 bp predicted BetI1-protected region of P*_betB_*, with forward oligonucleotides 5′-biotin-labeled (Table S2). P_29_ and P_30_ concentrations were elevated to 4 and 2.4 nM, respectively, as they were too short to be visible at regular DNA probe concentration in EMSA.

### Microscale thermophoresis-binding assay

The Large Volume Protein Labeling Kit RED-Tris-NTA 2nd Generation (NanoTemper Technologies GmbH) was used to label purified BetI2. Ligands were diluted in a range of concentrations. Diluted ligands were mixed with labeled BetI2 in buffer containing 1× PBS (pH 7.4) and 0.05% Tween-20 at 25°C. Mixed samples were loaded into MonolithTM NT.115 Series capillaries (NanoTemper Technologies GmbH), and thermophoresis was carried out on a Monolith NT.115 instrument (NanoTemper Technologies GmbH). The instrument was set to 60% LED power and “medium” microscale thermophoresis (MST) power for measurement. *K*_*d*_ values were obtained by fitting the MST data in the MO.Affinity Analysis software. The following ligands were used: DNA probe IGR, 0.5 nM–15.5 μM; DNA probe P1, 0.6 nM–19.5 μM; and DNA probe P2, 0.4 nM–14.5 μM.

### Bioinformatics

The identified *betI box* in *E. coli*, *P. aeruginosa*, and *S. meliloti* was aligned using ClustalW embedded in MEGA version 7.0. The weight matrix for the consensus sequences was generated using WebLogo (http://weblogo.berkeley.edu/). A maximum-likelihood phylogenetic tree based on protein sequences of BetI1 and BetI2 homologs that co-occurred in one strain (with genomes available in the NCBI) was constructed using MEGA version 7.0. Protein sequences of BetIs from *E. coli* K-12 substr. MG1655, *P. aeruginosa* PAO1, *A. baylyi* ADP1, and *Halomonas elongata* DSM 3043 were included to show the relationship of the two BetIs to these characterized BetIs. The tree topology was checked by 1,000 bootstrap replicates.

## RESULTS AND DISCUSSION

### Characterization of the *R. pomeroyi* DSS-3 *bet* cluster

The MRB model bacterium *R. pomeroyi* DSS-3 is able to metabolize COS and choline as carbon or nitrogen sources or for the synthesis of osmoprotectant GBT (20). The organization of *bet* genes in *R. pomeroyi* DSS-3 differs from those characterized in other bacteria, such as *E. coli*, *P. aeruginosa*, *A. baylyi*, *Halomonas elongata*, and *S. meliloti* (24, 26, 34, 35, 41) (Fig. 1). Both *R. pomeroyi* DSS-3 and *S. meliloti*, belonging to Alphaproteobacteria, contain *betC* in their *bet* clusters, indicating their ability to catabolize COS. In *R. pomeroyi* DSS-3, two extra genes with functions unrelated to COS/choline catabolism are present in the *bet* cluster, and the *betB* gene (SPO0084) is located far from the *bet* cluster. Notably, *R. pomeroyi* DSS-3 has two *betIs*, *betI1* (SPO1082) and *betI2* (SPO1086), in its *bet* cluster, while all other characterized *bet* clusters contain only one regulatory *betI* (Fig. 1). These two BetIs shared 33% protein sequence identity with each other and 27.4%–38.9% identity to other characterized BetIs (Table 1). *betI1* was shown to be co-transcribed with *betC* (SPO1083), while *betI2* was divergently transcribed from the co-transcribed *betT-betA* (SPO1087-1088) operon (Fig. S1). The existence of two BetIs implies a complicated regulatory pattern of COS and choline catabolism in *R. pomeroyi* DSS-3.

**TABLE 1:** Protein sequence identity between BetIs of *R. pomeroyi* DSS-3 and other characterized BetIs

Gene source	Sequence ID	Bet I1 (%)	BetI2 (%)
*Ruegeria pomeroyi* DSS-3	SPO1082	100	33.5
*Ruegeria pomeroyi* DSS-3	SPO1086	33.5	100
*Acinetobacter baylyi* ADP1	WP_004921794.1	27.9	27.4
*Escherichia coli* K-12 substr. MG1655	AAB18039.1	31.1	31.8
*Sinorhizobium meliloti* 102F34	AAC13370.1	38.9	29.1
*Pseudomonas aeruginosa* PAO1	WP_003096684.1	32.5	32.5
*Halomonas elongata* DSM 3043	WP_179152067.1	35.2	32.2

### COS and choline induce the transcription of *bet* genes except for *betI2*

**Fig 2:**
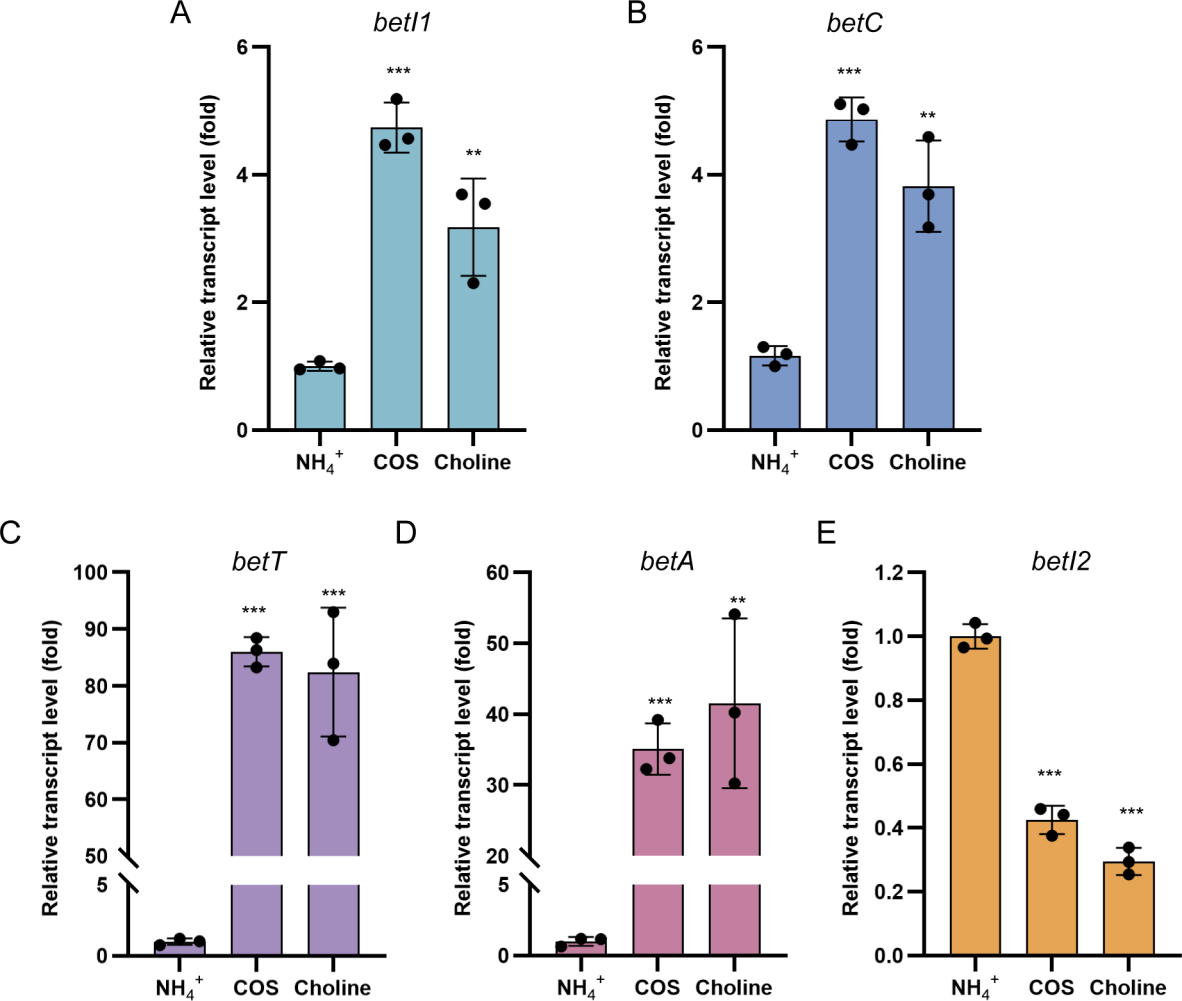
Relative transcript levels of *bet* genes in the presence of COS (2 mM) and choline (2 mM). The transcript level of each gene in the presence of NH_4_^+^ was used as the control. (**A**) Relative transcript level of *betI1*; (**B**) relative transcript level of *betC*; (**C**) relative transcript level of *betT*; (**D**) relative transcript level of *betA*; (**E**) relative transcript level of *betI2*. The error bar represents the standard deviation of triplicate experiments. A two-sided Student’s *t*-test was used to assess statistically significant differences (***P < 0.001; **P < 0.01; and *P < 0.05). All experiments were carried out at least three times.

Initially, the transcript levels of *bet* genes were monitored in wild-type *R. pomeroyi* cells grown on COS or choline (2 mM) as the sole nitrogen source. *betI1* and *betC* transcription were moderately upregulated (less than sixfold) by COS and choline (Fig. 2A and B). In contrast, COS and choline triggered more dramatic upregulation of *betT* and *betA* transcripts (35- to 85-fold) (Fig. 2C and D). It implied different regulatory patterns for the *betI1C* operon and *betTA* operon in response to COS and choline. Unexpectedly, *betI2*, sharing the intergenic region with the *betTA* operon, was significantly downregulated by COS and choline (Fig. 2E). Taken together, these distinct responses of *bet* genes to COS and choline indicate independent regulation of the *betI1C* operon and *betI2-betTA* divergon.

### BetI1 directly regulates the transcription of *betI1C* but not *betI2-betTA* divergon

**Fig 3:**
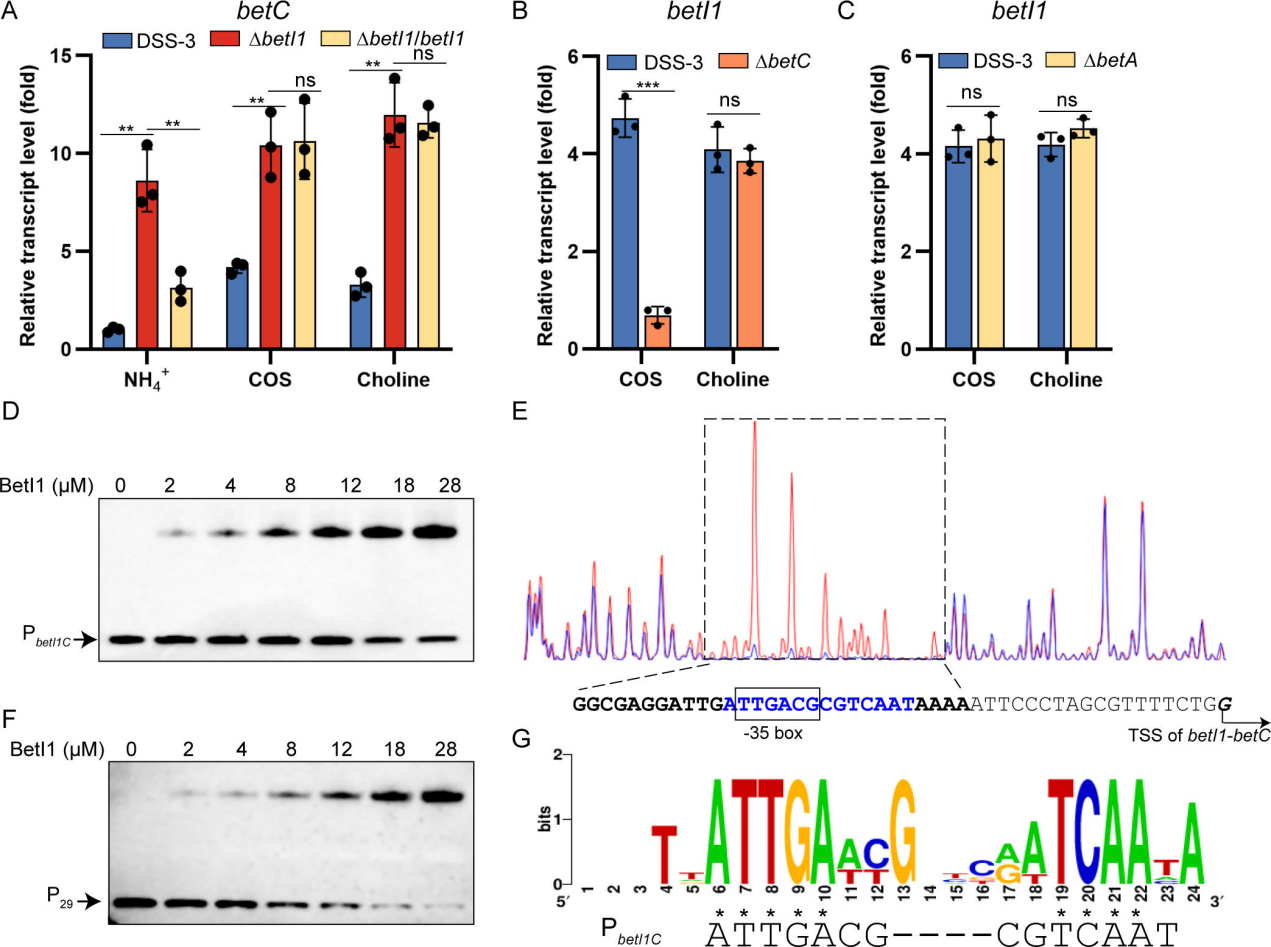
BetI1 directly regulates the transcription of *betI1-betC* with choline as effector. (**A**) Relative transcript level of *betC* in wild-type DSS-3, Δ*betI1,* and Δ*betI1*/*betI1* strains grown with 2 mM COS or choline compared to the NH_4_^+^ treatment. (B and C) Relative transcript level of *betI1* in wild-type DSS-3 and Δ*betC* (**B**) or Δ*betA* (**C**) strains grown with 2 mM COS or choline compared to the NH_4_^+^ treatment. (**D**) EMSAs of BetI1 titrated against 5′-biotin-labeled P*_betI1C_* (1 nM). (**E**) DNase I footprinting assay done on FAM-labeled probe P*_betI1C_* DNA in the presence (blue) and absence (red) of 2 µg BetI1. Partial promoter sequence of *betI1C* was shown underneath with the 29 bp BetI1-protected sequence in bold. The palindrome sequence in the BetI1-protected region is in blue. The predicted transcription start site (TSS) of *betI1C* is in bold and italics and indicated with an arrow. The −35 box of *betI1C* is framed. (**F**) EMSAs of BetI1 titrated against 5′-biotin-labeled P_29_ (4 nM), the 29 bp BetI1-protected sequence. (**G**) The consensus sequences of identified *betI boxes*. The palindrome of the P*_betI1C_* that matches the *betI box* consensus sequences was shown. A two-sided Student’s *t*-test was used to assess statistically significant differences (***P < 0.001; **P < 0.01; and ns, *P* > 0.05). All experiments were carried out at least three times.

To determine the regulatory role of the two BetIs on the transcription of the *bet* cluster, we generated Δ*betI1* and Δ*betI2* mutants. Next, the transcript levels of *bet* genes were investigated in the Δ*betI1* mutant. Since *betI1* and *betC* are co-transcribed (Fig. S1), the transcript level of *betC* was used to evaluate the effect of BetI1 on the *betI1C* operon. *betC* transcript level was elevated approximately ninefold in Δ*betI1* cells grown in medium containing NH_4_^+^ compared to wild-type cells (Fig. 3A). The *betC* transcript levels were comparable in Δ*betI1* with NH_4_^+^, COS, or choline, implying deregulated *betC* transcription. This deregulation in the Δ*betI1* grown on NH_4_^+^ was restored in the complemented mutant. In contrast, transcript levels of *betI2*, *betT*, and *betA* were indistinguishable between wild type and Δ*betI1* (Fig. S2A through C), implying that BetI1 does not participate in the transcription regulation of *betI2* and *betTA*. Thus, BetI1 specifically represses the transcription of *betI1C* in the *bet* cluster. Even though the two inserted genes (*SPO1084* and *SPO1085*) in the *bet* cluster (Fig. 1B) appeared unrelated to COS and choline catabolism, we investigated their regulation by BetI1. Neither COS nor choline induced *SPO1084* transcription (Fig. S2D). *SPO1084* transcript levels were comparable between wild-type and Δ*betI1* strains grown on NH_4_^+^ or COS (Fig. S2D). Notably, Δ*betI1* exhibited ~3.9-fold upregulation of *SPO1084* specifically under choline conditions compared to wild type (Fig. S2D). *SPO1085* transcript levels were decreased in wild type grown on COS and choline compared to those on NH_4_^+^ (Fig. S2E). A similar pattern was observed for *SPO1085*, with slight but significant deregulation (less than twofold) in Δ*betI1* grown with choline (Fig. S2E). These findings suggest that BetI1 may regulate *SPO1084* and *SPO1085* under choline conditions, though its regulatory mode differs from the canonical repression observed for *betI1C* in the *bet* cluster. Further studies are required to elucidate the functional implications and the mechanistic basis of this atypical regulation.

BetI is a choline-responsive repressor in *E. coli*, *S. meliloti*, and *Acinetobacter* spp. (34, 35, 38, 41). To identify the effector of BetI1, we measured the induction effects of COS and choline on *betI1* in Δ*betC* and Δ*betA* mutant strains, which blocked the COS and choline catabolism at the initial step, respectively. COS induction was totally abolished by knocking out *betC* (Fig. 3B), implying that the derepression by COS requires its conversion to choline or some downstream catabolite(s). In contrast, the induction effect of COS was unaffected when *betA* was absent (Fig. 3C), indicating that choline was the *bona fide* effector of BetI1. Consistently, the induction effects of choline on *betI1* were comparable between the wild-type DSS-3 and Δ*betA* (Fig. 3C), supporting choline as the effector of BetI1.

Electrophoretic mobility shift assay was performed to determine the interaction between purified BetI1 and 5′-biotin-labeled promoter region of *betI1C* (P*_betI1C_*). A significant shift was detected, which was enhanced by increased BetI1 concentration (Fig. 3D). DNase I footprinting analysis on P*_betI1C_* showed strong protection of a 29 bp region (Fig. 3E). This 29 bp DNA, termed P_29_, exhibited a strong BetI1 concentration-dependent EMSA shift (Fig. 3F). A perfect palindrome (ATTGACG*CGTCAAT; asterisk indicates symmetry center) was identified in the 29 bp BetI1-protected region (Fig. 3E), matching the *betI box* consensus (Fig. 3G) (37, 40). This implies that BetI1 in *R. pomeroyi* DSS-3 resembles the previously characterized BetIs in other bacteria. The identified 29 bp BetI1-protected region encompassed the −35 box of *betI1C* predicted using the BPROM program (50), implying that BetI1 functions as a repressor by blocking the transcription initiation of *betI1C*.

### BetI2 directly regulates the transcription of *betTA* but not *betI1C*

**Fig 4:**
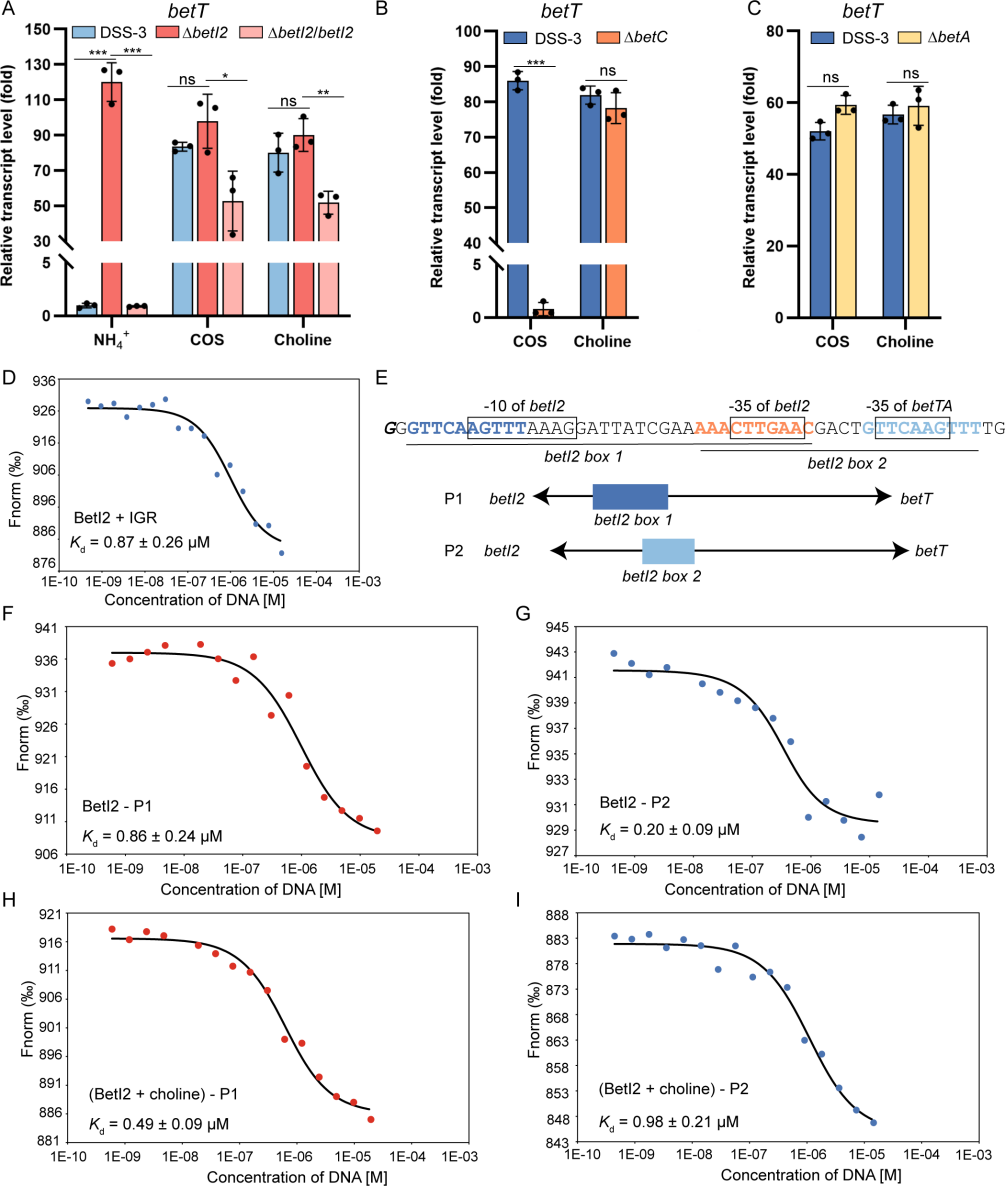
BetI2 directly regulates the transcription of *betTA* with choline as an effector. (**A**) Relative transcript level of *betT* in wild-type DSS-3, Δ*betI2,* and Δ*betI2*/*betI2* strains grown with 2 mM COS or choline compared to the NH_4_^+^ treatment. (**B** and **C**) Relative transcript level of *betT* in wild-type DSS-3 and Δ*betC* (**B**) or Δ*betA* (**C**) strains grown with 2 mM COS or choline compared to the NH_4_^+^ treatment. (**D**) MST analysis of BetI2 binding to DNA probe IGR. BetI2 had a *K*_*d*_ of 0.87 ± 0.26 µM for IGR. (**E**) The partial sequence of IGR containing the two overlapped palindromes (in bold) and a schematic of the DNA probes used in MST assays. The two palindromes were underlined and termed *betI2 box 1* and *betI2 box 2*, respectively. Flanking sequences of the two palindromes are shown in dark blue and light blue, respectively. Shared sequence of the two palindromes is in orange. −10 and −35 boxes are framed. P1, probe IGR variant containing only *betI2 box 1*; P2, probe IGR variant containing only *betI2 box 2*. (**F**–**I**) MST analysis of BetI2 binding to DNA probe P1 or P2 with or without the presence of choline. A two-sided Student’s *t*-test was used to assess statistically significant differences (***P < 0.001; **P < 0.01; *P < 0.05; and ns, *P* > 0.05). All experiments were carried out at least three times.

The transcript levels of *betT* and *betA* were constitutively upregulated in Δ*betI2* (Fig. 4A; Fig. S3A), implying that BetI2 was the transcription repressor of the *betTA* operon. In contrast, the absence of *betI2* did not affect the transcript levels of *betI1* and *betC* (Fig. S3B and C), demonstrating that BetI2 specifically represses *betTA* transcription. Similarly, we investigated the regulatory roles of BetI2 in controlling the two inserted genes within the *bet* cluster. The transcript levels of both *SPO1084* and *SPO1085* were indistinguishable between the wild-type and Δ*betI2* strains under all tested conditions (Fig. S3D and E), demonstrating that these genes were not regulated by BetI2. The induction effect of COS on *betT* was lost in Δ*betC* (Fig. 4B) but was maintained in Δ*betA* (Fig. 4C), indicating that choline is also the effector of BetI2.

Interaction between purified BetI2 and the intergenic region of *betI2* and *betTA* was detected by EMSA (Fig. S3F) and MST, yielding a *K*_*d*_ value of 0.87 ± 0.26 µM (Fig. 4D). To determine the exact BetI2-binding site, we analyzed the IGR sequence. Two palindromes (*betI2 box 1* and *betI2 box 2*) sharing flanking sequence AAACTTGAAC were identified (Fig. 4E). Note that both *betI2 boxes* did not match the consensus sequence of *betI box* (Fig. 3G).

Based on the promoters of *betI2* and *betTA* operons predicted by BPROM (50), the *betI2 box 1* spans the −10 and −35 of *betI2*, while *betI2 box 2* overlaps both −35 of *betI2* and *betTA* (Fig. 4E). This implies that the binding of BetI2 to *betI2 box 1* only hinders the transcription initiation of *betI2*, while *betI2 box 2* binding affects both. Since COS and choline repress the transcription of *betI2* but induce the transcription of *betTA* (Fig. 2C through E), we suspect that the presence of effector choline modulates the binding affinity of two partially overlapped *betI2 boxes*. To test this, the IGR probe variants were constructed, containing only *betI2 box 1* (P1) and only *betI2 box 2* (P2). The affinities of BetI2 to the IGR probe variants with or without choline were determined by MST. Interactions between BetI2 to P1 and P2 without choline were detected, indicating that BetI2 can bind to either palindrome (Fig. 4F and G). However, the *K*_*d*_ value of BetI2 to P1 was slightly higher (approximately fourfold) than that to P2, implying BetI2 favors *betI2 box 2* over *betI2 box 1* when no effector is present. Based on the location of *betI2 box 2*, the binding of BetI2 on *betI2 box 2* hinders the initiation of both *betI2* and *betTA* operons in the absence of choline. Consistently, when choline was supplemented, the affinity of BetI2 to P1 was a little higher than to P2 (approximately twofold) (Fig. 4H and I), indicating that *betI2 box 1* was the preferred DNA binding site of BetI2. In this situation, BetI2 binding to DNA only blocks the transcription initiation of *betI2* and leaves the *betTA* promoter accessible. This explained that COS and choline induce the transcription of *betTA* but repress the transcription of *betI2*.

### BetI1, but not BetI2, regulates the transcription of *betB*

**Fig 5:**
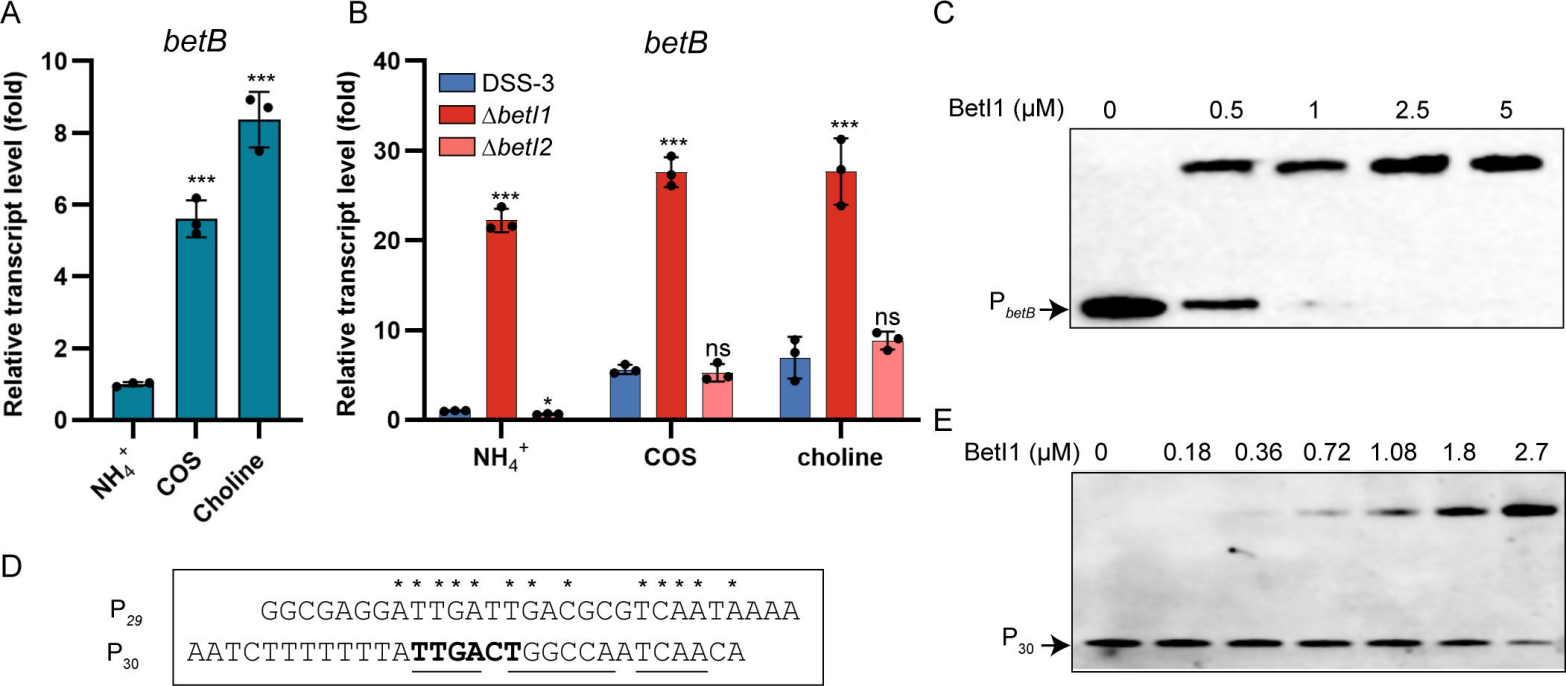
*betB* belongs to the BetI1 regulon. (**A**) Relative transcript level of *betB* in wild-type DSS-3 grown with 2 mM COS or choline compared to the NH_4_^+^ treatment. (**B**) Relative transcript of *betB* in the wild-type DSS-3, Δ*betI1*, and Δ*betI2* strains grown with 2 mM COS or choline compared to the NH_4_^+^ treatment. (**C**) EMSAs of BetI1 titrated against biotin-5′-labeled P*_betB_* (1 nM). (**D**) Sequence alignment of P_29_ (the BetI-protected region in the *betI1C* promoter) and P_30_ (the partial sequence of the *betB* promoter containing an imperfect palindrome, underlined), resembling the *betI1 box*. Conserved nucleotides between P_29_ and P_30_ are indicated by asterisks. The predicted −35 box of *betB* is highlighted in bold. (**E**) EMSAs of BetI1 titrated against biotin-5′-labeled P_30_ (2.4 nM). A two-sided Student’s *t*-test was used to assess statistically significant differences (***P < 0.001; *P < 0.05; and ns, *P* > 0.05). All experiments were carried out at least three times.

The transcript fold changes of *betB* (<10-fold), which is located out of the *bet* cluster in *R. pomeroyi* DSS-3 (Fig. 1), were comparable to those of *betI1C* when grown on COS or choline compared to NH_4_^+^ treatment (Fig. 5A, 2A, and B). This implies that *betB* may be controlled by BetI1, like the *betI1C* operon. To test this, we measured the *betB* transcript level in wild-type DSS-3, Δ*betI1*, and Δ*betI2* mutant strains. As expected, *betB* transcript level was constitutively upregulated in Δ*betI1*, while the absence of *betI2* barely had an effect (Fig. 5B). This indicated that BetI1, rather than BetI2, regulated the transcription of *betB*. BetI1 concentration-dependent EMSA shifts were detected when incubated with the promoter region of *betB* (Fig. 5C). Since BetI1 directly regulates the transcription of both *betI1C* operon and *betB*, the BetI1 binding site in the promoter of *betB* should resemble the verified *betI1 box* in the promoter of *betI1C*. As expected, an imperfect palindrome, which matches the consensus sequences of the *betI1 box*, was identified in the promoter of *betB* (Fig. 5D). Similar to the *betI1 box* in the promoter of *betI1C* (Fig. 3C), this imperfect palindrome also encompasses the predicted −35 box of *betB* (Fig. 5D). EMSA shifts of a 30 bp DNA probe encompassing the palindromic sequence of the *betB* promoter (P_30_) were enhanced with the increasing BetI1 (Fig. 5E). These results indicate that BetI1 simultaneously controls the transcription of *betI1C* and *betB*.

### Transcription of *betI2-betTA* divergon is regulated by osmotic stress

**Fig 6:**
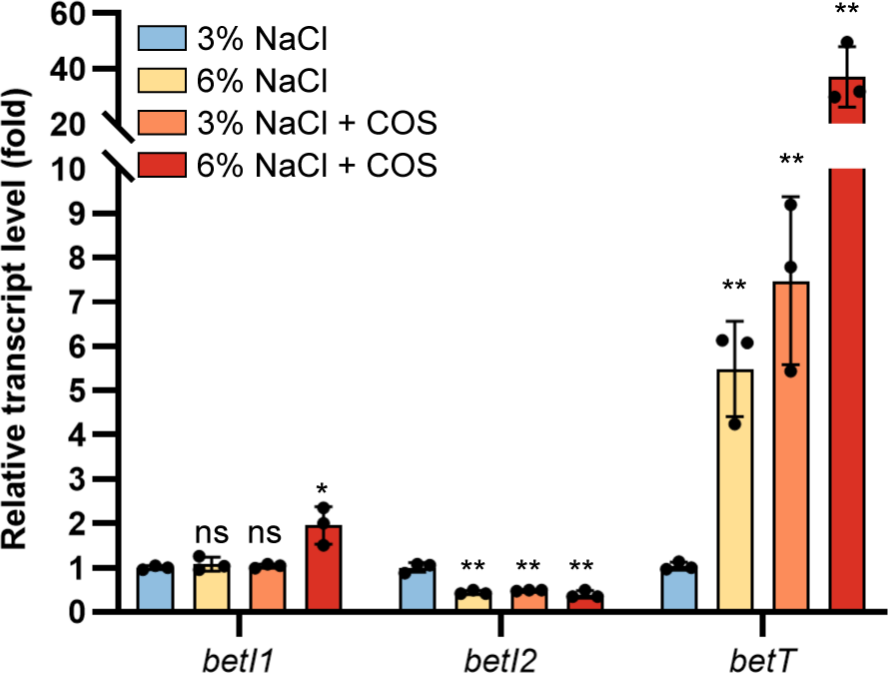
*betI2-betTA* divergon responds to osmotic stress. Relative transcript levels of *betI1*, *betI2*, and *betT* in wild-type DSS-3 grown in 3% NaCl or 6% NaCl minimal medium in the presence of COS or not (**P < 0.01; *P < 0.05; and ns, *P* > 0.05). All experiments were carried out at least three times.

In addition to serving as carbon and nitrogen sources, COS *per se* could act as a compatible osmolyte (2, 7) and also serve as the source of the bacterial compatible solute GBT. To address whether the *bet* cluster is regulated by salinity as well, the transcript levels of *betI1*, *betI2,* and *betT* were monitored to show the response of *betI1C*, *betI2*, and *betTA* operons to osmotic stress with or without COS. The transcript level of *betI1* in 6% NaCl, the highest NaCl concentration that *R. pomeroyi* DSS-3 could tolerate (Fig. S4A), was indistinguishable from that in 3% NaCl, the regular NaCl concentration for marine bacteria (Fig. 6). It implies that the *betI1C* operon was not induced by osmotic stress. This observation aligns with the finding in Alphaproteobacteria *S. meliloti*, where *betI* from the *betICBA* operon did not respond to osmotic stress (41). The presence of COS (not as a nitrogen source) in 3% NaCl did not trigger the upregulation of *betI1* (Fig. 6), supporting the role of BetIC in the utilization of COS as a nitrogen source. However, 6% NaCl plus COS led to a significant but weak upregulation (less than twofold) of *betI1* transcripts. These results indicate that BetI1 and BetC are not for osmoadaptation.

In contrast, high salinity (6% NaCl) significantly decreased the transcript of *betI2* while enhancing the transcript of *betT* (Fig. 6). This resembles the divergent effect of COS or choline on the *betI2-betTA* divergon when used as nitrogen sources (Fig. 2C through E). The presence of COS in 3% NaCl also induced the transcript of *betT* (~7.5-fold) (Fig. 6). However, it was not comparable to the dramatic upregulation of *betT* (~85-fold) observed when COS was used as the sole nitrogen source (Fig. 2C). The response of structural genes *betC* and *betTA* (Fig. 2B through D and 6) indicated that the *bet* cluster is dramatically induced by COS only when it is the only available nitrogen source. The combination of high salinity (6% NaCl) and COS caused the greatest upregulation of *betT* (~37-fold). These data are in accord with those in *A. baylyi* and *Acinetobacter nosocomialis* (35, 36). Previous studies reported that osmotic stress induces the transcription of *betIs* in *E. coli*, *P. aeruginosa*, and *A. baylyi* (23, 24, 28), promoting choline uptake (via BetT) and oxidation (via BetBA). However, in *R. pomeroyi* DSS-3, high salinity repressed *betI2*, thereby augmenting the deregulation of *betTA* in addition to the effector binding of BetI2. The presence of intracellular choline subsequently induces the transcription of *betB* by releasing BetI1 from its promoter, enabling catabolism of the intermediate betaine aldehyde to GBT to cope with osmotic stress. In addition, *R. pomeroyi* DSS-3 Δ*betT* exhibited reduced growth when COS was the sole nitrogen source (Fig. S4B), indicating that BetT also participates in the uptake of COS even though some unknown COS transporter(s) exist (Fig. 1A). Thus, under high salinity conditions with the presence of COS, the decrease of *betI2* enhances the transcription of *betTA,* accelerating COS and choline uptake and catabolism. This will help the strain to cope with osmotic stress.

### Existence of two BetIs is widespread in Alphaproteobacteria

**Fig 7:**
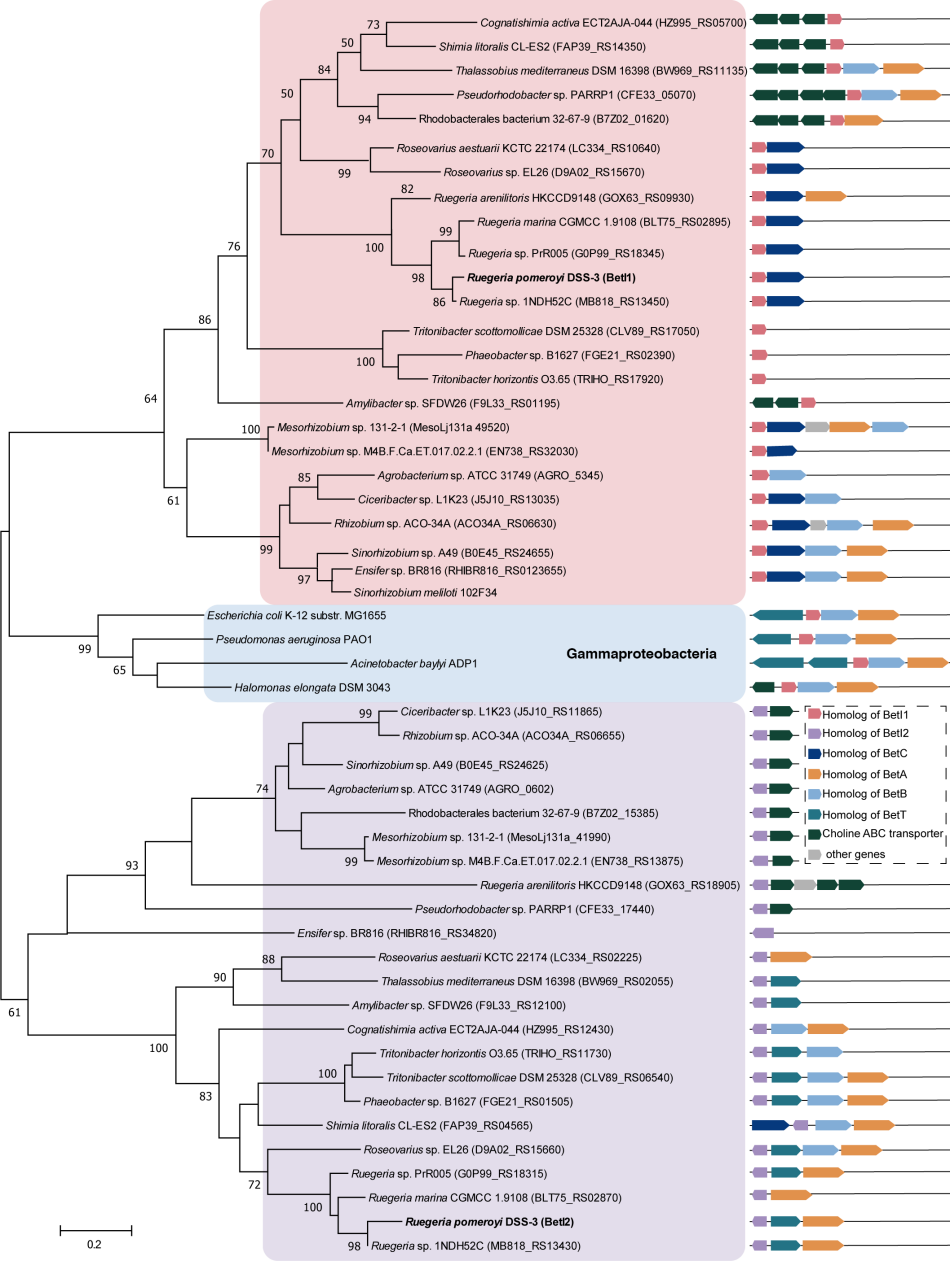
Maximum-likelihood phylogenetic tree of BetI1 and BetI2 homologs and the genomic arrangement of *betI* genes. Except for the distinct branch consisting of Gammaproteobacteria (shaded in light blue), strains in the phylogenetic tree containing homologs of both BetI1 and BetI2 are all Alphaproteobacteria. *R. pomeroyi* DSS-3 is shown in bold. Homologs of BetI1 are highlighted in pink. Homologs of BetI2 are shaded in light purple. Bootstrap values (percentage of 1,000 replications) are shown at the nodes of the tree, and values <50% are omitted. Bar, 0.2 substitutions per amino acid position.

Next, the NCBI genome database was screened for bacteria possessing homologs of the two BetIs. Bacteria containing both BetIs were exclusively Alphaproteobacteria, mainly Rhodobacterales, with some Hyphomicrobiales (Table S3). In the phylogenetic tree based on these BetIs, homologs of BetI1 and BetI2 from *R. pomeroyi* DSS-3 formed distinct branches, respectively (Fig. 7). Alphaproteobacteria *S. meliloti* 102F34 BetI was located in the BetI1 homologs branch (Fig. 7). Characterized BetIs from *E. coli* K-12 substr. MG1655, *P. aeruginosa* PAO1, *A. baylyi* ADP1, and *H. elongata* DSM 3043—all belonging to Gammaproteobacteria—formed a distinct branch (Fig. 7). This suggests divergent evolution of *betI*s across taxonomic groups.

In addition, the genomic neighborhoods of the two *betI*s are diverse (Fig. 7). It is noteworthy that *betC* is almost exclusively adjacent to *betI1*, except in *Shimia litoralis* CL-ES2, implying a conserved role for BetI1 in regulating the initial step of COS catabolism. Even though some *betI1* homologs were adjacent to the choline ABC transporter operon *choXWV* (51, 52), *betI2* homologs were more frequently clustered with choline transporters (BetT and ChoXWV) or orphan substrate binding protein (ChoX). It suggests that bacteria are more prone to adopt BetI2 to modulate the transport of choline.

The coexistence of two BetIs with different regulatory targets in COS and choline catabolism, along with distinct responses to osmotic stress, indicates a novel regulatory mechanism adopted by abundant Alphaproteobacteria. Since both BetIs sense the same effector (choline)—whether directly imported or derived from COS catabolism—this simultaneously upregulates all *bet* genes involved in choline assimilation. When exposed to high salinity, it is urgent to produce the potent osmolyte GBT. Under this condition, the osmo-responsive *betI2-betTA* divergon is activated. The repression of *betI2* by high salinity and the subsequent import of choline will further enhance the uptake of GBT precursor choline and its catabolism by BetA. It seems irrational to adopt BetI1 to control the transcription of *betB*, which catalyzes the BetA product into GBT. However, *betB* is not the only betaine aldehyde dehydrogenase since its knockout did not abolish the growth of *R. pomeroyi* DSS-3 on choline as the sole carbon source (20). Thus, the dramatically accelerated choline uptake and its subsequent catabolism enhance GBT production, enabling rapid adaptation to osmotic stress.

### Conclusions

COS and choline are ubiquitous in the environment, and their catabolism, mediated by the *bet* genes, serves as both a source of the potent osmoprotectant GBT and nutrients for bacteria. This study elucidates a novel regulatory mechanism of COS and choline catabolism by two BetIs. The two homologous BetIs, termed BetI1 and BetI2, respectively, control the distinct steps of COS and choline catabolism in the MRB model strain *R. pomeroyi* DSS-3 (Fig. 1A). BetI1 deregulates the transcription of *betC* and *betB* in response to the presence of effector choline, but not to high salinity. *R. pomeroyi* DSS-3 modulates the binding affinities of BetI2 to the two partially overlapping *betI2 boxes*, enabling divergent regulation of *betI2* and *betTA* transcription in response to both the effector choline and high salinity. The existence of two BetIs is identified in Alphaproteobacteria, mainly Rhodobacterales and Hyphomicrobiales. This study provides a novel insight into the catabolism of COS and choline by abundant bacteria as a nutrient or to cope with osmotic stress.

## Data Availability

The data underlying this article are available in the article and in its online supplemental material. Additional data will be made available on request.

## SUPPLEMENTAL MATERIAL

Supplemental material aem.00333-25-s0001.docx Tables S1 to S3; Fig. S1 to S4.

ASM does not own the copyrights to Supplemental Material that may be linked to, or accessed through, an article. The authors have granted ASM a non-exclusive, world-wide license to publish the Supplemental Material files. Please contact the corresponding author directly for reuse.
