# Supplementary material for: Novel regulatory mechanism of choline-O-sulfate and choline catabolism by two BetIs in Alphaproteobacteria
Source: Appl Environ Microbiol. 2025 Aug 13;91(9):e00333-25. doi: 10.1128/aem.00333-25 (PMC12442346; doi:10.1128/aem.00333-25)
Supplement: Supplemental material — Tables S1 to S3; Fig. S1 to S4. [file aem.00333-25-s0001.docx]

**Table S1** Bacterial strains and plasmids used in this study

| Strains/plasmids | Description/use | Reference or source |
| --- | --- | --- |
| *E. coli* WM3064 | Donor strain for conjugation, Δ*dapA* | W. Metcalf, UIUC |
| *E. coli* DH5α | Gene cloning host strain | Vazyme |
| *E. coli* BL21 (DE3) | Recombinant protein expression host strain | Vazyme |
| *Ruegeria pomeroyi* DSS-3 | type strain | Laboratory preservation |
| Δ*betI1* | *betI1*-deletion mutant derived from *R. pomeroyi* DSS-3 | This study |
| Δ*betI2* | *BetI2*-deletion mutant derived from *R. pomeroyi* DSS-3 | This study |
| Δ*betA* | *betA*-deletion mutant derived from *R. pomeroyi* DSS-3 | This study |
| Δ*betC* | *betC*-deletion mutant derived from *R. pomeroyi* DSS-3 | This study |
| Δ*betT* | *betT*-deletion mutant derived from *R. pomeroyi* DSS-3 | This study |
| Δ*betI1*/*betI1* | Complementation of Δ*betI1* | This study |
| Δ*betI2*/*betI2* | Complementation of Δ*betI2* | This study |
| Δ*betT*/*betT* | Complementation of Δ*betT* | This study |
| pHGM01 | Suicide vector used for gene deletion, Gm^r^ | Jin et al, 2013 |
| pHG101 | Promoterless broad-host vector, Km^r^ | Wu et al, 2011 |
| pHG101-*betI1* | Complementation vector of Δ*betI1* | This study |
| pHG101-*betI2* | Complementation vector of Δ*betI2* | This study |
| pHG101-*betT* | Complementation vector of Δ*betI2* | This study |
| pET28a (+) | Km^r^, His-tagged protein expression Vector | Novagen |
| pET28a-*betI1* | pET28a carrying the coding region of *betI1* | This study |
| pET28a-*betI2* | pET28a carrying the coding region of *betI2* | This study |

**Table S2** Primers used in this study

| Oligonucleotides | Sequence (5’→3’) |
| --- | --- |
| Mutagenesis |  |
| *betI1*-5’O | GGGGACAAGTTTGTACAAAAAAGCAGGCTGATGATCCCCTTTGCGGTGG |
| *betI1*-5’I | AAGTGCGCCTAATCGCGTAGCTGCTATCGTCGCCTTGACG |
| *betI1*-3’I | CTACGCGATTAGGCGCACTTACCCTGATCGAGGCTGGTAG |
| *betI1*-3’O | GGGGACCACTTTGTACAAGAAAGCTGGGTCCTCTTGCCGGGTGGTTTC |
| *betI2*-5’O | GGGGACAAGTTTGTACAAAAAAGCAGGCTGCATAGATCACATCCGCACC |
| *betI2*-5’I | AAGTGCGCCTAATCGCGTAGGCGCGCTGACCGAAATG |
| *betI2*-3’I | CTACGCGATTAGGCGCACTTATGCGTTTGCGTCTCATGC |
| *betI2*-3’O | GGGGACCACTTTGTACAAGAAAGCTGGGTCGCCGATCTCGGGAATGTAG |
| *betC*-5’O | GGGGACAAGTTTGTACAAAAAAGCAGGCTTTGATCTGTCCCCCATCACAC |
| *betC*-5’I | AAGTGCGCCTAATCGCGTAGGATCAGGATATTGGGTCGGGTC |
| *betC*-3’I | CTACGCGATTAGGCGCACTTTAGGCAAGACGGTTCTCTTCG |
| *betC*-3’O | GGGGACCACTTTGTACAAGAAAGCTGGGTAACGGGTAAGGTCCGGTAAC |
| *betA*-5’O | GGGGACAAGTTTGTACAAAAAAGCAGGCTTGACCGATTACCACGCCTTC |
| *betA*-5’I | AAGTGCGCCTAATCGCGTAGAATCCGCTTCCATCCGTCTG |
| *betA*-3’I | CTACGCGATTAGGCGCACTTCGCACCCGATTTGATCCCT |
| *betA*-3’O | GGGGACCACTTTGTACAAGAAAGCTGGGTAGCTGGTCTATGCTGACAACC |
| *betT*-5’O | GGGGACAAGTTTGTACAAAAAAGCAGGCT GTTTCGAGCACGGGAATTAG |
| *betT-*5’I | AAGTGCGCCTAATCGCGTAGTTCAGGATCATGGTCCCTCC |
| *betT*-3’I | CTACGCGATTAGGCGCACTTCCTTTGTCATCAACTCGCTG |
| *betT*-3’O | GGGGACCACTTTGTACAAGAAAGCTGGGTGCAGGATATCGCAATTCTCG |
| Complementation |  |
| pHG101-*betI1*-F | GCCCCGGGTGGTACCTGAATTCGACACGCCGGTTGTGCAATC |
| pHG101-*betI1*-R | GGTACTAGTAGGATCCCCTCGAGGGGTCGGGTTCATCTACCAG |
| pHG101-*betI2*-F | GCCCCGGGTGGTACCTGAATTCCGCGAGCGGGTTGGCAAAG |
| pHG101-*betI2*-R | GGTACTAGTAGGATCCCCTCGAGCTCGCGCCTTGTCACCCC |
| pHG101-*betT-*F | GCCCCGGGTGGTACCTGAATTCGTGTCTTTGCCTTGGCCAGC |
| pHG101-*betT*-R | GGTACTAGTAGGATCCCCTCGAGTTGTGTCAATGCACGCTTTCGA |
| RT-qPCR |  |
| qRT-*betI1*-F | ATCTATTGGCGCTGAGGAGT |
| qRT-*betI1*-R | CTGCTATCGTCGCCTTGA |
| qRT-*betI2*-F | TGCATCAGCGAGGATTCGG |
| qRT-*betI2*-R | GCGCATGGTCTCTTCCAT |
| qRT-*betC*-F | TGATCCTGATGGTGGATCAG |
| qRT-*betC*-R | GCATTGGCAAACCGGGTCG |
| qRT-*betT*-F | TTTGGTCTGGTGGCCTTTTG |
| qRT-*betT*-R | TCAGCCCGACATCCAGGC |
| qRT-*betA*-F | CGGATTTTGTCATCGTGGGG |
| qRT-*betA*-R | CGGGCATCTGGATGAACGG |
| qRT-*betB*-F | GAAGCGAGCCATTTCATCAAT |
| qRT-*betB*-R | CCTCGAGCGCCTGATCGAT |
| RT-PCR |  |
| RT-*betI1C*-F | TTCGCGCATTCCTGTTTCAC |
| RT-*betI1C*-R | CGCAGGTGATGGGCATAAGT |
| RT-*betTA*-F | TGATCCGGCTTTACACCGAC |
| RT-*betTA*-R | CGGTTGTTCAGATGCGGTTC |
| EMSA DNA probe |  |
| Probe-P*_betI1C_* -F | Biotin-TGTCCCCCATCACACGCAT |
| Probe-P*_betI1C_* -R | CCCACTCCTCAGCGCCAAT |
| Probe-P_29_ -F | Biotin-GGCGAGGATTGATTGACGCGTCAATAAAA |
| Probe-P_29_ -R | TTTTATTGACGCGTCAATCAATCCTCGCC |
| Probe-P*_betI2_*-F | Biotin-GGCCATATTGGCCAATCGG |
| Probe-P*_betI2_*-R | ATCATGGTCCCTCCCTGGGT |
| Probe-P*_betB_*-F | Biotin-CGAGATCCAAGCCGACAACAT |
| Probe-P*_betB_*-R | TTCATGATGCGTCCTTTGTGC |
| Probe-P_30_-F | Biotin-AATCTTTTTTTATTGACTGGCCAATCAACA |
| Probe-P_30_-R | TGTTGATTGGCCAGTCAATAAAAAAAGATT |
| Heterogenous expression |  |
| pET28a-*betI1*-F | GGTGCCGCGCGGCAGCCATATGCCGAAAATCGGAATGGAACCG |
| pET28a-*betI1*-R | GGTGGTGGTGGTGGTGCTCGAGTCATCTACCAGCCTCGATCAG |
| pET28a-*betI2*-F | GGTGCCGCGCGGCAGCCATATGAGACGCAAACGCATTCGGG |
| pET28a -*betI2*-R | GGTGGTGGTGGTGGTGCTCGAGTCACCCCCGCTCCAGCAG |

**Table S3** Genomic organization of *bet* clusters in the representative strains with two BetIs.

| **Strain** | **Taxonomy** | ***betI1*** | ***betI2*** | ***betC*** | ***betA*** | ***betB*** | ***betT*** | ***choXWV*** |
| --- | --- | --- | --- | --- | --- | --- | --- | --- |
| *Cognatishimia active* ECT2AJA-044 | Alphaproteobacteria; [Rhodobacterales](https://www.ncbi.nlm.nih.gov/Taxonomy/Browser/wwwtax.cgi?mode=Undef&id=204455&lvl=3&lin=f&keep=1&srchmode=1&unlock) | + | + |  | + | + |  | + |
| *Shimia litoralis* CL-ES2 | Alphaproteobacteria; [Rhodobacterales](https://www.ncbi.nlm.nih.gov/Taxonomy/Browser/wwwtax.cgi?mode=Undef&id=204455&lvl=3&lin=f&keep=1&srchmode=1&unlock) | + | + | + | + | + |  | + |
| *Thalassobius mediterraneus* DSM 16398 | Alphaproteobacteria; [Rhodobacterales](https://www.ncbi.nlm.nih.gov/Taxonomy/Browser/wwwtax.cgi?mode=Undef&id=204455&lvl=3&lin=f&keep=1&srchmode=1&unlock) | + | + |  | + | + |  | + |
| *Pseudorhodobacter* sp. PARRP1 | Alphaproteobacteria; [Rhodobacterales](https://www.ncbi.nlm.nih.gov/Taxonomy/Browser/wwwtax.cgi?mode=Undef&id=204455&lvl=3&lin=f&keep=1&srchmode=1&unlock) | + | + |  | + | + |  | + |
| Rhodobacterales bacterium 32-67-9 | Alphaproteobacteria; [Rhodobacterales](https://www.ncbi.nlm.nih.gov/Taxonomy/Browser/wwwtax.cgi?mode=Undef&id=204455&lvl=3&lin=f&keep=1&srchmode=1&unlock) | + | + |  | + |  |  | + |
| *Roseovarius aestuarii* KCTC 22174 | Alphaproteobacteria; [Rhodobacterales](https://www.ncbi.nlm.nih.gov/Taxonomy/Browser/wwwtax.cgi?mode=Undef&id=204455&lvl=3&lin=f&keep=1&srchmode=1&unlock) | + | + | + | + |  |  |  |
| *Roseovarius* sp. EL26 | Alphaproteobacteria; [Rhodobacterales](https://www.ncbi.nlm.nih.gov/Taxonomy/Browser/wwwtax.cgi?mode=Undef&id=204455&lvl=3&lin=f&keep=1&srchmode=1&unlock) | + | + | + | + | + | + |  |
| *Ruegeria arenilitoris* HKCCD9148 | Alphaproteobacteria; [Rhodobacterales](https://www.ncbi.nlm.nih.gov/Taxonomy/Browser/wwwtax.cgi?mode=Undef&id=204455&lvl=3&lin=f&keep=1&srchmode=1&unlock) | + | + | + | + |  |  | + |
| *Ruegeria marina* CGMCC 1.9108 | Alphaproteobacteria; [Rhodobacterales](https://www.ncbi.nlm.nih.gov/Taxonomy/Browser/wwwtax.cgi?mode=Undef&id=204455&lvl=3&lin=f&keep=1&srchmode=1&unlock) | + | + | + | + |  |  |  |
| *Ruegeria* sp. PrR005 | Alphaproteobacteria; [Rhodobacterales](https://www.ncbi.nlm.nih.gov/Taxonomy/Browser/wwwtax.cgi?mode=Undef&id=204455&lvl=3&lin=f&keep=1&srchmode=1&unlock) | + | + | + | + |  | + |  |
| R*uegeria pomeroyi* DSS-3 | Alphaproteobacteria; [Rhodobacterales](https://www.ncbi.nlm.nih.gov/Taxonomy/Browser/wwwtax.cgi?mode=Undef&id=204455&lvl=3&lin=f&keep=1&srchmode=1&unlock) | + | + | + | + |  | + |  |
| *Ruegeria* sp. 1NDH52C | Alphaproteobacteria; [Rhodobacterales](https://www.ncbi.nlm.nih.gov/Taxonomy/Browser/wwwtax.cgi?mode=Undef&id=204455&lvl=3&lin=f&keep=1&srchmode=1&unlock) | + | + | + | + |  | + |  |
| *Tritonibacter scottomollicae* DSM 25328 | Alphaproteobacteria; [Rhodobacterales](https://www.ncbi.nlm.nih.gov/Taxonomy/Browser/wwwtax.cgi?mode=Undef&id=204455&lvl=3&lin=f&keep=1&srchmode=1&unlock) | + | + |  | + | + | + |  |
| *Phaeobacter* sp. B1627 | Alphaproteobacteria; [Rhodobacterales](https://www.ncbi.nlm.nih.gov/Taxonomy/Browser/wwwtax.cgi?mode=Undef&id=204455&lvl=3&lin=f&keep=1&srchmode=1&unlock) | + | + |  | + | + | + |  |
| *Tritonibacter horizontis* O3.65 | Alphaproteobacteria; [Rhodobacterales](https://www.ncbi.nlm.nih.gov/Taxonomy/Browser/wwwtax.cgi?mode=Undef&id=204455&lvl=3&lin=f&keep=1&srchmode=1&unlock) | + | + |  |  | + | + |  |
| *Amylibacter* sp. SFDW26 | Alphaproteobacteria; [Rhodobacterales](https://www.ncbi.nlm.nih.gov/Taxonomy/Browser/wwwtax.cgi?mode=Undef&id=204455&lvl=3&lin=f&keep=1&srchmode=1&unlock) | + | + |  |  |  | + | + |
| *Mesorhizobium* sp. 131-2-1 | Alphaproteobacteria; [Hyphomicrobiales](https://www.ncbi.nlm.nih.gov/Taxonomy/Browser/wwwtax.cgi?mode=Undef&id=356&lvl=3&lin=f&keep=1&srchmode=1&unlock) | + | + | + | + | + |  | + |
| *Mesorhizobium* sp. M4B.F.Ca.et.017.02.2.1 | Alphaproteobacteria; [Hyphomicrobiales](https://www.ncbi.nlm.nih.gov/Taxonomy/Browser/wwwtax.cgi?mode=Undef&id=356&lvl=3&lin=f&keep=1&srchmode=1&unlock) | + | + | + |  |  |  | + |
| *Agrobacterium* sp. ATCC 31749 | Alphaproteobacteria; [Hyphomicrobiales](https://www.ncbi.nlm.nih.gov/Taxonomy/Browser/wwwtax.cgi?mode=Undef&id=356&lvl=3&lin=f&keep=1&srchmode=1&unlock) | + | + |  | + |  |  | + |
| *Ciceribacter* sp. L1K23 | Alphaproteobacteria; [Hyphomicrobiales](https://www.ncbi.nlm.nih.gov/Taxonomy/Browser/wwwtax.cgi?mode=Undef&id=356&lvl=3&lin=f&keep=1&srchmode=1&unlock) | + | + | + |  | + |  | + |
| *Rhizobium* sp. ACO-34A | Alphaproteobacteria; [Hyphomicrobiales](https://www.ncbi.nlm.nih.gov/Taxonomy/Browser/wwwtax.cgi?mode=Undef&id=356&lvl=3&lin=f&keep=1&srchmode=1&unlock) | + | + | + | + | + |  | + |
| *Sinorhizobium* sp. A49 | Alphaproteobacteria; [Hyphomicrobiales](https://www.ncbi.nlm.nih.gov/Taxonomy/Browser/wwwtax.cgi?mode=Undef&id=356&lvl=3&lin=f&keep=1&srchmode=1&unlock) | + | + | + | + | + |  | + |
| *Ensifer* sp. BR816 | Alphaproteobacteria; [Hyphomicrobiales](https://www.ncbi.nlm.nih.gov/Taxonomy/Browser/wwwtax.cgi?mode=Undef&id=356&lvl=3&lin=f&keep=1&srchmode=1&unlock) | + | + | + | + | + |  |  |

**Supplementary Figures**


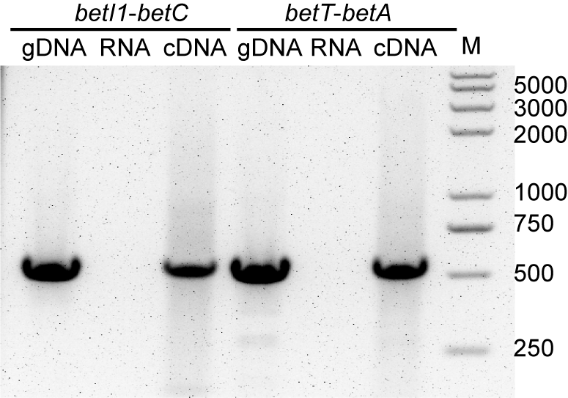


**Fig. S1** **Co-transcription of *betI1*-*betC* and *betT*-*betA*.** RT-PCR of *betI1*-*betC* and *betT*-*betA*. PCR amplification of DNA fragments ranging from *betI1* to *betC* and ranging from *betT* to *betA* with genome DNA (gDNA), RNA and cDNA as template, respectively.


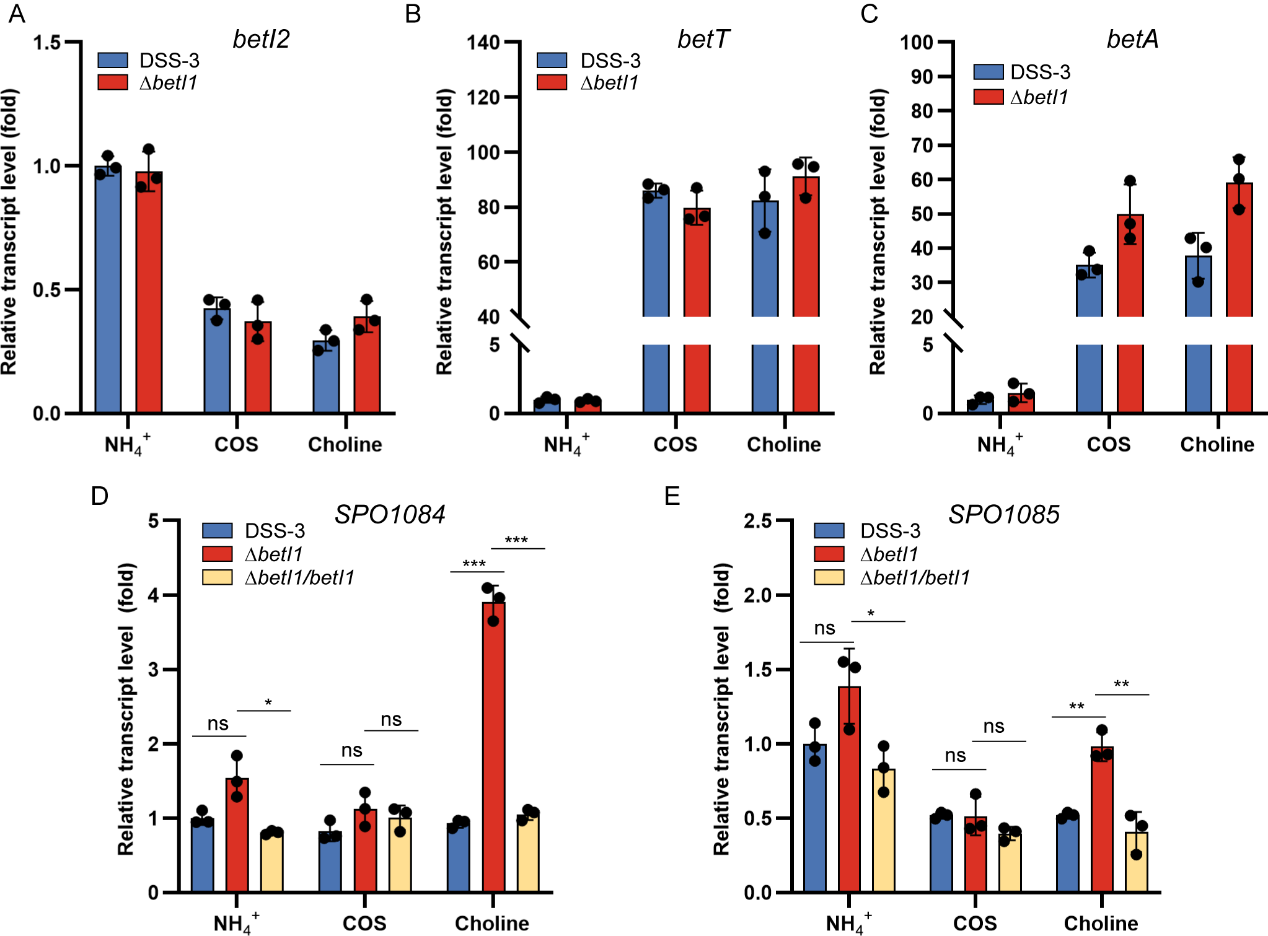


**Fig. S2 Divergon *betI2* and *betTA* did not belong to the BetI1 regulon.** Relative transcript levels of *betI2* (A), *betT* (B), *betA* (C), *SPO1084* (D), and *SPO1085* (E) in wild type DSS-3 and Δ*betI1* strains grown with 2 mM COS or choline compared to the NH_4_^+^ treatment. All experiments were carried out at least three times.

**
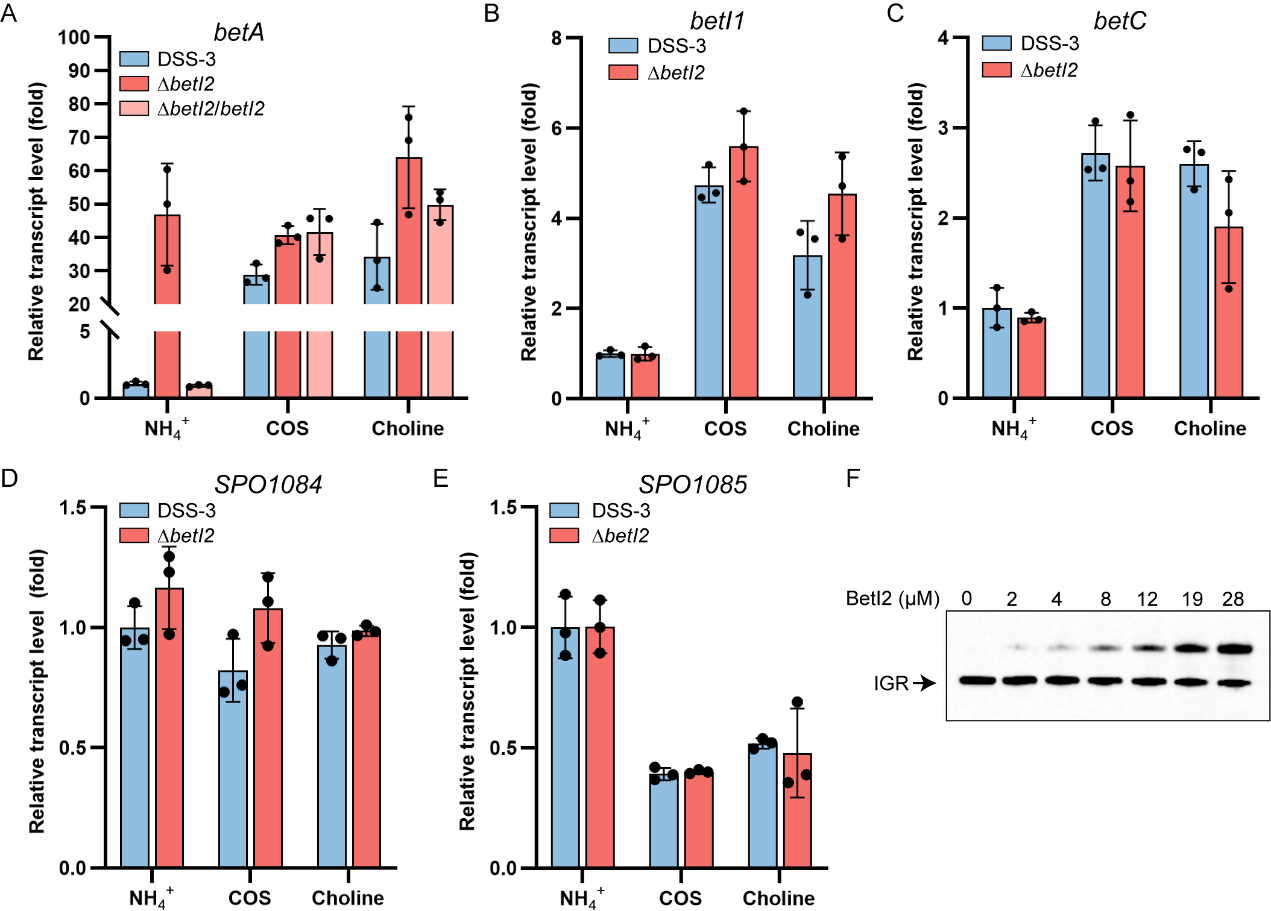
**

**Fig. S3 BetI2 regulates the transcription of *betTA* but not *betI1C*.** A. Relative transcript level of *betA* in wild type DSS-3, Δ*betI2*, and Δ*betI2/betI2* strain grown with 2 mM COS or choline compared to the NH_4_^+^ treatment. B, C, D, and E. Relative transcript level of *betI1* (B), *betC* (C), *SPO1084* (D), and *SPO1085* (E) in wild type DSS-3 and Δ*betI2* strain grown with 2 mM COS or choline compared to the NH_4_^+^ treatment. F. EMSA of BetI2 titrated against 5’-biotin-labled intergenic region (IGR) of *betI2* and *betTA* operons (20 nM). All experiments were carried out at least three times.


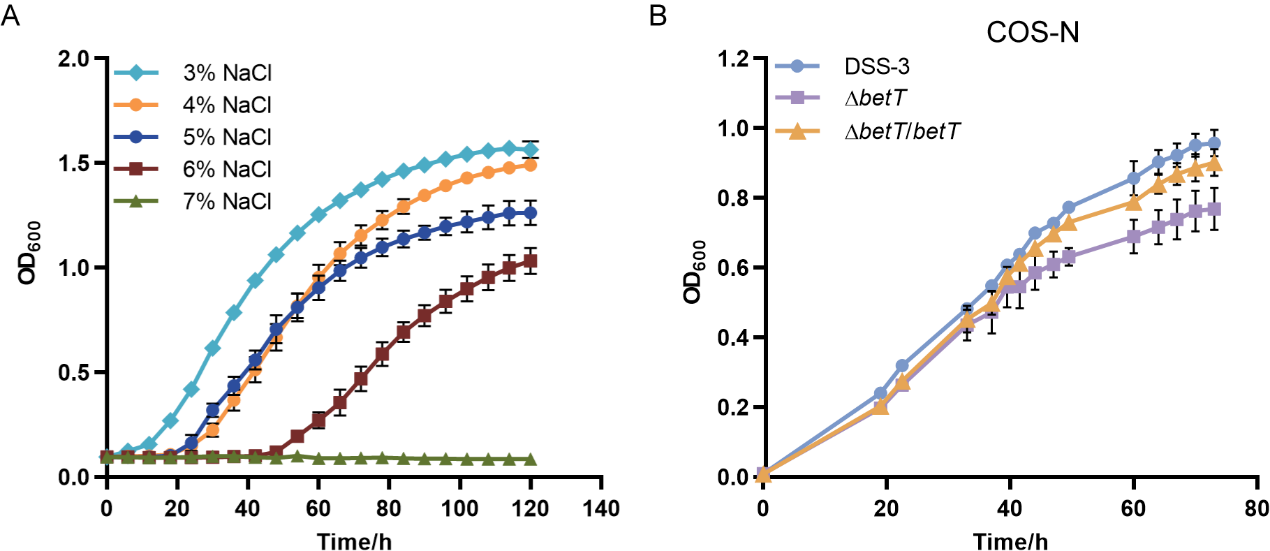


**Fig. S4** Salinity tolerance of *R. pomeroyi* DSS-3 and the involvement of BetT in COS uptake. A. Growth of *R. pomeroyi* DSS-3 in the presence of different concentrations of NaCl. B. Growth of wild type DSS-3, Δ*betT*, and Δ*betT*/*betT* in minimal medium with COS (2 mM) as nitrogen source. All experiments were carried out at least three times.
